# Dermoscopic Appearance of an Annular Subacute Cutaneous Lupus Erythematosus

**DOI:** 10.5826/dpc.1102a13

**Published:** 2021-03-08

**Authors:** Biswanath Behera, Rashmi Kumari, Debasis Gochhait, Pavithra Ayyanar

**Affiliations:** 1 Department of Dermatology and Venereology, AIIMS, Bhubaneswar, India; 2 Department of Dermatology, Venereology and Leprology, JIPMER, Puducherry, India; 3 Department of Pathology, JIPMER, Puducherry, India; 4 Department of Pathology, AIIMS, Bhubaneswar, India

**Keywords:** annular SCLE, subacute cutaneous lupus erythematosus, SCLE, dermoscopy

## Introduction

Subacute cutaneous lupus erythematosus (SCLE) is characterized mainly by cutaneous disease and usually has a good prognosis. The presence of nonscarring papulosquamous and annular polycyclic lesions are the hallmark of the disease. Although the diagnosis is a straightforward task, at times, it can be confused with other inflammatory dermatoses. There are limited dermoscopic features described for SCLE [1].

## Case Presentation

**Figure 1:**
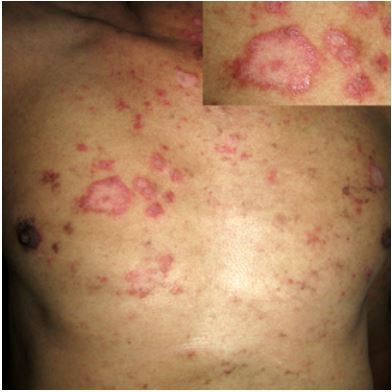
Multiple erythematous scaly annular plaques over the chest. Inset shows peripheral rim of scaling.

A 40-year-old man with skin phototype IV presented with a 1-month history of multiple reddish skin lesions over the anterior chest, neck, and upper back. It was associated with a history of photosensitivity and on-and-off joint pain. He denied any history of prior drug intake. Cutaneous examination revealed multiple erythematous, scaly papules and annular plaques. A few of the plaques showed a peripheral rim of white scales (Figure 1). The differential diagnoses of SCLE, psoriasis, and petaloid seborrheic dermatitis were considered.

**Figure 2:**
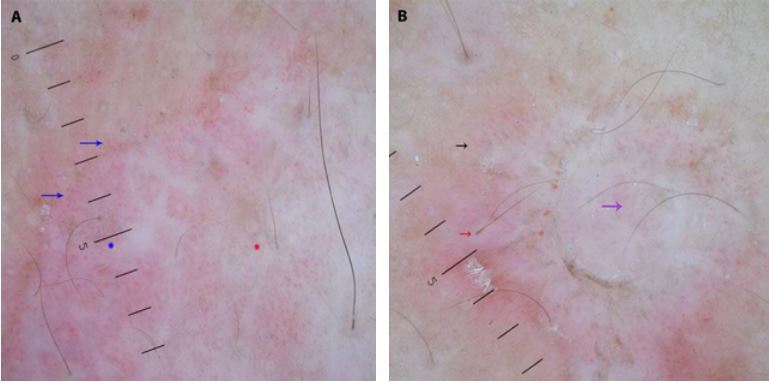
(A, B) Dermoscopy (Heine Delta20 Dermatoscope; magnification ×10)) shows a white to reddish-white homogenous area, patchy clustered brown to blue-gray dots (blue asterisk), blue-gray peppering (red asterisk), scales, and a mixed vascular pattern, comprised of linear, hairpin (black arrow) and glomerular (blue arrows) vessels. Prominent white homogenous area around blood vessels (purple arrow) and normal looking hair follicles (red arrow).

**Figure 3:**
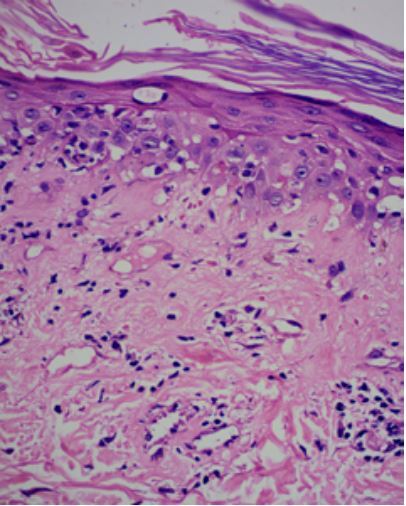
Histology shows hyperkeratotic atrophic epidermis, basal vacuolar degeneration, cytoid body, melanin incontinence, and upper dermal dilated blood vessels (H&E, ×400).

Dermoscopic examination showed a white to reddish-white homogenous area, patchy clustered brown to blue-gray dots and peppering (dots without circumscription), scales, and a mixed vascular pattern comprised of linear, hairpin, dotted, and glomerular vessels. The white homogenous area surrounded, or become prominent, around the blood vessels and hair follicles (Figure 2). There was no follicular plugging or dilatation, and the hair follicles within the lesions appeared to be normal. Laboratory investigations revealed mild anemia (hemoglobin 11.2gm/dl) and positive antinuclear and anti-Ro antibodies. Histopathology showed hyperkeratosis, a focal atrophic epidermis, basal vacuolar degeneration, pigmentary incontinence, and upper dermal telangiectatic blood vessels (Figure 3). Immunohistochemistry showed a linear deposition of immunoreactant IgG and C3 along the dermoepidermal junction. The diagnosis of SCLE was made. The patient was advised in strict sun protection and started with tablet hydroxychloroquine 200 mg (4 mg/kg per day) once daily, topical mometasone ointment, and sunscreen.

Two constant dermoscopic features, whitish scales and a mixed vascular pattern over a pinkish, or reddish background, were reported in a case series of SCLE. The scales were located either diffusely or at the periphery. The vessels included linear, linear irregular, branching, and sparsely distributed dotted vessels [1]. A similar pattern was observed in the index case, except for the presence of a prominent white homogenous area and brown to blue-gray dots/peppering.

Dermoscopy can be a useful tool to differentiate annular SCLE from its main differential diagnoses that include psoriasis, that is characterized by the presence of regularly distributed dotted vessels and white scales on a light to dull red background; pityriasis rosea, that usually shows peripheral white scales (collarette sign) and patchy dotted vessels; and granuloma annulare, that demonstrates a whitish to yellow-orange area along with varying morphology vessels. In contrast to SCLE, discoid lupus erythematosus is dominated by follicular plugging, dilated follicles, a perifollicular whitish halo, and a monomorphic vascular pattern, a feature that can be helpful to distinguish extrafacial discoid lupus erythematosus from SCLE [2].

## Conclusion

In conclusion, a dermoscopy pattern, white to reddish-white homogenous area with brown to blue-gray dots/peppering and mixed vascular pattern, can be useful to diagnose and differentiate annular SCLE from its clinical mimics.
